# Supplementary material for: Degeneracy measures in biologically plausible random Boolean networks
Source: BMC Bioinformatics. 2022 Feb 14;23:71. doi: 10.1186/s12859-022-04601-5 (PMC8845291; doi:10.1186/s12859-022-04601-5)
Supplement: Supplementary file 1 — Additional file 1. Supplementary Materials. [file 12859_2022_4601_MOESM1_ESM.docx]

# Supplementary Information

**S1 Text 1. *RBN Model Explanations***

**S1 Text 2. *cBN Simulation Platform***

**S1 Text 3. *cBN Model Specifications***

**S1 Figure 1. A toy network with Boolean functions and translation into SDE.**

**S1 Text 4. *Simulations with Network Size N = 100.***

**SI Figure 2. Simulation results for N = 100.**

***RBN Model Explanations***

For RBN simulations (available from: https://github.com/basakkcgl/RBNsims) we have generated random networks where each node is connected to other two nodes via Boolean rules with operators: *AND, OR, AND NOT*, *OR NOT*, *NOT AND*, *NOT OR*, *NOT AND NOT*, *NOT OR NOT*, *XOR*. For nodes that have only one incoming edge, there are two rules can that take place: COPY or, NOT. For *COPY*, the node is directly connected to another node without a logical effect. All the operators have equal probability to be assigned. The pseudocode for rule-assignment is provided below.

LIST rules for one-edge connection = [NOT, COPY]

LIST rules for two-edges connection = [AND, OR, AND NOT, OR NOT, NOT AND, NOT OR, NOT AND NOT, NOT OR NOT, XOR]

edges = INT RANDOM (either 1 or 2)

IF edges = = 1:

rule = RANDOM choice from (LIST rules for one -edge connection)

ENDIF

IF edges = = 2:

rule = RANDOM choice from (LIST rules for two-edges connection)

ENDIF

***cBN Simulation Platform***

To generate synthetic gene expression data, we ran simulations on the open source BoolODE pipeline (available from: <https://github.com/Murali-group/BoolODE>) V0.1 release. The following adjustments were made:

1. The BoolODE pipeline calls a text file that has a Boolean model as input. First, the model is translated into stochastic differential equations (SDEs) with added noise terms, after which numerical simulations (that result in a stochastic time course) are run [68]. As input files, we generated random Boolean networks that are converted into text files and these files are called from BoolODE to generate datasets as described above.
2. To work with SDEs, BoolODE uses a version of Euler integration that has a noise parameter *c.* We arbitrarily set the noise parameter to 0.3 to introduce stochasticity. This integration method is referred to be a similar function in MATLAB ode45 (for source code, see <https://murali-group.github.io/Beeline/_modules/src/BoolODE.html#simulateModel>).
3. In cases where simulations of network activity attenuates (goes to zero), the simulation was initiated with different starting conditions. If network activity still attenuated, it was assumed to be the result of that specific network configuration, and the network was processed with the last conditions of random initialization to further analysis.
4. Additional simulations with 100.000 RBNs are conducted to test the effect of multistability on degeneracy measures. Results support the null hypothesis: the number of attractors in a network does not have a significant effect on degeneracy measures (one-way ANOVA, F (5,99974) = 0.48, p = 0.7888).

***cBN Model Specifications***

For cBN simulations we have generated random networks where each node is connected to other two nodes via Boolean rules with operators: *OR, AND NOT.* In pilot simulations we have conducted with many rules inclusive (such as AND, NOT AND, OR NOT), we have not observed a difference in terms of mutual information and degeneracy measures. The choice for these operators that bind 2 genes (edges = 2) is made for automating the process to input files to BoolODE simulations to achieve efficient computing time.

For nodes that have only one incoming edge, there are two rules that can take place: COPY or, NOT. For *COPY*, the node is directly connected to another node without a logical effect. The pseudocode for rule-assignment is provided below.

LIST rules for one -edge connection = [NOT, COPY]

LIST rules for two-edges connection = [AND NOT, OR]

edges = INT RANDOM (either 1 or 2)

IF edges = = 1:

rule = RANDOM choice from (LIST rules for one -edge connection)

ENDIF

IF edges = = 2:

rule = RANDOM choice from (LIST rules for two-edges connection)

ENDIF

Each random Boolean network is converted into an equivalent SDE that is a continuous model of gene regulation. The conversion steps are (using the same framework in GeneNetWeaver [68, 70]) as follows:

1. Each node in the network is assigned to a ‘gene’ variable representing the level of messenger RNA expression and a ‘protein’ variable representing the amount of transcription factors.
2. The level of transcription factor is determined by a model that takes into account mRNA transcription and degradation rates, as well as protein translation and degradation rates. Transcription and translation are counterbalanced by the degradation of the mRNA and protein pools.
3. Transcription rate depends on the affinity of gene’s promoter to the transcription factor. The probability of each binding-configuration is computed. Then, the efficiency of transcription activation by a specific configuration of bound regulators is calculated (with added noise term to introduce stochasticity). The cooperative effects of regulator binding are set by the parameters of Hill threshold and Hill coefficient.

In this study, we have preserved default model specifications for gene regulation in the V01 release of BoolODE pipeline. Perturbations are injected to genes (not to proteins) as Gaussian variance with *mean = 0* and *standard deviation = 0.01* at each time step of the simulations.

The gene regulatory model parameters are preserved as in the original mathematical model that is used in Pratapa et al. [68]. The parameters and their default values are as follows:

mRNA transcription rate (*m*) = 20

mRNA degradation rate (*l_x_*) = 10

Protein translation rate (*r*) = 10

Protein degradation rate (*l_p_*) = 1

Hill threshold (*k*) = 10

Hill coefficient (*n*) = 10

The states of each gene are determined by Boolean functions with input states representing the activity of the regulatory factors (incoming edges). All possible effects from incoming edges are converted into parameter α_p_ where “p” stands for the parent node (source of the edge). The value of the parameter α_p_ is the output of the Boolean function relating to the activity (/state, ON or OFF) of the target gene (child node) and the state of the regulatory factor (parent node).


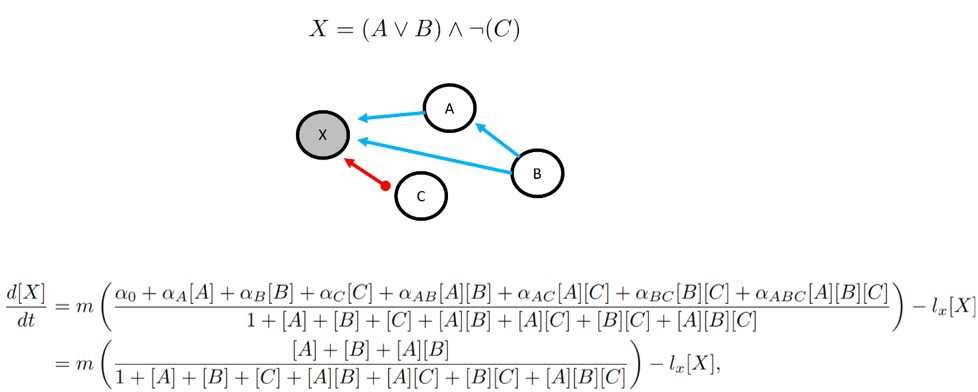


**Supplementary Information Figure 1.** **A toy network with Boolean functions and translation into SDE.** The Boolean function (*top*) for the target gene X defined by the configuration of the incoming edges. Incoming edges show the regulatory control over the target gene, and are represented as arrows (*middle*), where upregulation correspond to blue arrows and red arrow represent inhibitory control. The logical function (*top*) and its equivalent SDE for gene X (*bottom*) is retrieved from Pratapa et al. [68].

***Simulations with Network Size N = 100***

RBNs with total number of nodes 100 are generated with same procedures as described in SI Text 1. The entropy and partial degeneracy are calculated by the same method described in manuscript Methods section.


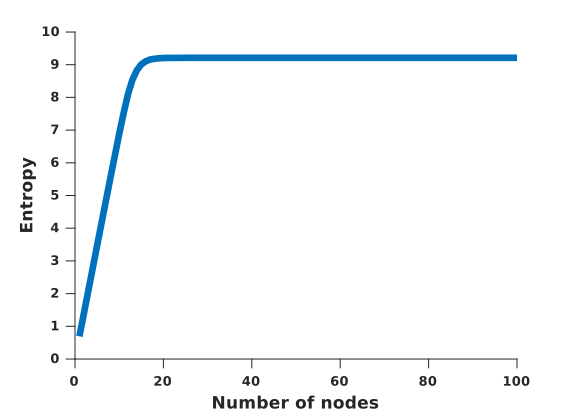

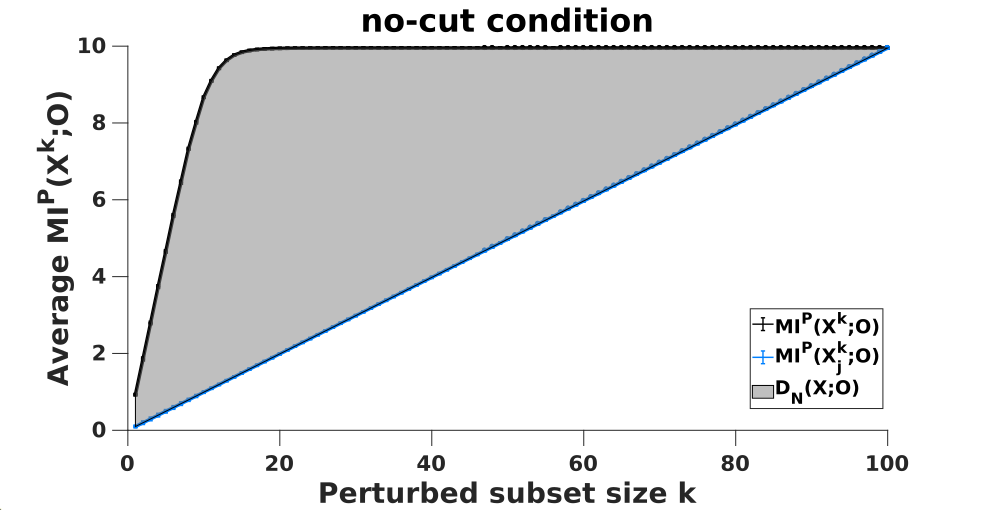


**Supplementary Information Figure 2. Measures for entropy and degeneracy in RBNs with network size 100.** The entropy (top) and degeneracy (bottom) calculated for conditions in which the number of perturbed units is greater than around 20 reaches an upper bound.

**References**

68. Pratapa A, Jalihal AP, Law JN, Bharadwaj A, Murali TM. Benchmarking algorithms for gene regulatory network inference from single-cell transcriptomic data. Nat Methods. 2020 Feb;17(2):147–54.

70. Schaffter T, Marbach D, Floreano D. GeneNetWeaver: in silico benchmark generation and performance profiling of network inference methods. Bioinforma Oxf Engl. 2011 Aug 15;27(16):2263–70.
